# One year of Communications Medicine

**DOI:** 10.1038/s43856-022-00136-1

**Published:** 2022-06-30

## Abstract

We are celebrating a year since *Communications Medicine* published its first articles. Here, we reflect on how far the journal has come, and share the goals that we will work towards in coming years.

It is time to celebrate the achievements of the last twelve months, and to thank our authors, reviewers, readers, editorial board members, and all other colleagues for their trust and support during our early days as a journal.

We launched in June 2021 with the aim of becoming an inclusive open-access journal for high-quality research, reviews, and commentary of interest to specialist audiences across all clinical, translational, and public health research fields. In the last year, we have received over 500 submissions from authors in 47 different countries, and published over 100 articles, both research and commentary, across many areas of our broad scope. These have already accrued more than 200 citations and have been downloaded over 300000 times, which is testament to how well the communities we work with have responded to this new journal.

“During this year we remained adaptable, innovative, and true to the ethos presented at launch—to be an inclusive open-access medical journal that aims to facilitate and disseminate discovery that will promote health for all and improve the lives of those experiencing or living with disease.”

Our first year has been undoubtedly dominated by the COVID-19 pandemic. It was a unique time to launch a journal covering medicine and public health, and a privilege to contribute to the topical discussions and shared knowledge on SARS-CoV-2/COVID-19. As a sign of the times, many of our articles focus on SARS-CoV-2/COVID-19, from novel ways to detect SARS-CoV-2, to understanding the clinical pathology of COVID-19 and assessing prognosis, to forecasting how the pandemic might evolve in different scenarios, or identifying factors that influence vaccine hesitancy. We have also published on other topics of interest for public health and epidemiology, such as antimicrobial resistance, and in many areas of clinical and translational research, such as oncology, digital pathology, and neurology. Our editors and editorial board members have put together a collection of article highlights that showcases some of the fascinating work we’ve published since launch.

Speaking of editors, our editorial team has grown over the year. We welcomed two new in-house editors and our editorial board went from 9 members at launch to 27 (and growing), who now cover a broader range of research fields and medical specialties. In keeping with our aim to be an inclusive journal with a diverse editorial board, 11 countries are now represented in our board and we have achieved an almost even gender balance.

During this year we remained adaptable, innovative, and true to the ethos presented at launch—to be an inclusive open-access medical journal that aims to facilitate and disseminate discovery that will promote health for all and improve the lives of those experiencing or living with disease. We’ve introduced a new article type, the Viewpoint, to facilitate our aim of fostering discussion and collaboration between fields and giving a voice to all stakeholders; you can read more about it here. We have published our first Q&As during Pride month to give voice to important health-related topics for the LGBTQ + community. We have also started to consider Registered Reports, an article format that is peer reviewed before and after the analyses have been performed. Acceptance is not dependent on the results obtained, so this article has the potential to not only ensure a more robust study design, but also to minimise publication and research bias. We have also joined a pilot Registered Reports Funding Partnership with Cancer Research UK (CRUK), alongside 12 other journals and supported by researchers at the University of Bristol; you can find more about this pilot here.

Looking forward, our goals are threefold. First, to become a trusted open access journal for all communities across the breadth of our scope for work with appeal to a specialist audience. Second, to contribute to building trust in medical research, by supporting public and patient participation, and by setting a high standard for research reporting, transparency and reproducibility. Third, to continue fostering dialogue across the different communities in our scope, while keeping diversity, equity, and inclusion at the centre of what we do. We will collaborate closely with our editorial board and we will use every opportunity to innovate in ways that advance these goals.

In the near future, we will focus on learning from the communities that we already work with, and on expanding to work with new communities. To support this, we will surface important areas of discussion in our commentary articles, and we will launch the first *Communications Medicine* call for papers for a guest-edited collection—watch this space! We will also be partnering with other Nature Portfolio journals in their calls for papers, the first example of which is being part of the *Nature Medicine* call for papers on clinical cancer research alongside *Nature Communications*.

We are very interested in hearing from all clinicians and scientists working in areas under our broad scope, with a special emphasis on work that is aligned with the UN Sustainable Development Goals (SDGs), SDG3 and SDG5 in particular, that we are committed to help facilitate. If you would like to learn more about *Communications Medicine* as a potential venue to publish your research, please do get in touch. You can request a ‘Meet the Editors’ session, email one of us, or find us on Twitter (@CommsMedicine).

Our first year has been a thrilling journey, and we cannot wait to see what the second one has in store!

